# Evaluation of Anti-Convulsive Properties of Aqueous Kava Extract on Zebrafish Using the PTZ-Induced Seizure Model

**DOI:** 10.3390/brainsci10080541

**Published:** 2020-08-11

**Authors:** Yogini Jaiswal, Mohd. Farooq Shaikh, Ilya Wang, Yanning Yong, Vanessa Lin Lin Lee, Leonard Williams

**Affiliations:** 1 Center for Excellence in Post-Harvest Technologies, The North Carolina Research Campus, 500 Laureate Way, Kannapolis, NC 28081, USA; 2 Neuropharmacology Research Laboratory, Jeffrey Cheah School of Medicine & Health Sciences, Monash University Malaysia, Bandar Sunway 47500, Malaysia; farooq.shaikh@monash.edu (M.F.S.); yongyanning@gmail.com (Y.Y.); Vanessa.Lee1@monash.edu (V.L.L.L); 3 Beaver College of Health Sciences, Appalachian State University, 287 Rivers Street, Boone, NC 28608, USA; wangij@appstate.edu; 4 School of Science, Monash University Malaysia, Bandar Sunway 47500, Malaysia

**Keywords:** Kava (*Piper methysticum*), anti-convulsive activity, PTZ-induced seizure model, zebrafish

## Abstract

Kava roots have been extensively studied in clinical trials as potential candidate anti-anxiety drugs. However, anti-convulsive properties of various tissues of stems of Kava have not been reported to date. The objective of the study was to evaluate the anti-convulsive potential of aqueous extracts prepared from specific tissues of Kava (*Piper methysticum*) stems in zebrafish, using the PTZ-induced seizure model. The potency of each extract was compared in terms of the intensity of seizure scores and onset time after pre-treating the zebrafish before the PTZ challenge. The results indicate that aqueous extract of Kava stems without peel after 45 min of pre-treatment exhibited anti-convulsive potential at the dose of 50 mg/L. This study provides evidence to the anti-convulsive properties of peeled Kava stems and its potential for investigation and design of candidate anti-convulsive drugs.

## 1. Introduction

*Piper methysticum* (commonly known as Kava), is a plant native to the South Pacific islands. The aqueous Kava root decoctions are commonly consumed as a ceremonial drink in the South Pacific [1]. It has growing demand and popularity in the global herbal drug market for its various herbal formulations and as a stress-relieving beverage. It is used as a recreational beverage in various other countries and is sold in various forms in Kava bars. These beverages are popular for their anti-anxiety, mood-stabilizing, sedative, and anti-depressant effects. The Kava lactones are reported to be the major constituents responsible for its psychoactive properties. Some reports indicate inconclusive cases of hepatotoxicity caused by intake of Kava, and such toxicity effects are suggested to be due to drug interactions with kava, improper dosage, or use of misidentified plant or tissue parts [2,3]. However, evidence of the toxicity of Kava roots itself is not supported by substantial scientific pieces of evidence. In its traditional use, the aerial parts of the roots and the stem are not recommended for consumption [4,5,6]. Studies reported to date have investigated roots, leaves, or stem peelings of various varieties. However, to date, no studies report a systematic tissue-specific analysis of Kava stems.

Like any other plant product available in the herbal drug market, Kava products also face the challenges associated with inadequate quality control, adulteration, contamination, substitution with cheaper varieties, and misidentification of plant parts. Although stem peelings have been reported to cause hepatotoxicity, no reports have studied the anti-convulsive effects and toxicity of other tissues of stems, which could be present as adulterants or as misidentified parts used in the preparation of herbal Kava formulations. Methysticin is one of the major kavalactones that contribute to neuroprotective effects in ischemia. In combination with dihydromethysticin and memantine, it is reported to significantly reduce the area of brain infarction in mice [7]. Kava extracts are reported to provide symptomatic relief of anxiety in clinical trials and in mice models, a characteristic that deviates from the usual mediation by benzodiazepine on the GABA_A_ receptor binding site [8,9,10].

The issues discussed above highlight the significance of the study of Kava extracts in the identification of a candidate anti-convulsant agent that may alleviate the severity of seizures without the need to suffer the cognitive and behavioral side effects that are associated with currently available anti-epileptic drugs (AED). This article reports the first study for evaluation of toxicity and anti-convulsive effect of extracts of various tissues of Kava stems in the zebrafish.

## 2. Materials and Methods

### 2.1. Extraction and Plant Material

*Piper methysticum* (Kava) stems collected from plants over three years of maturity were commercially obtained from Haridaya enterprises Ltd. (Waiyevo, Fiji). The whole stems, stem peels only, and stems with no peels were obtained and powdered to coarse size and used for extraction. For aqueous extracts, 50 g of stem powder for each sample was extracted by refluxing with 200 mL of distilled water. The resulting supernatant was filtered and freeze-dried to obtain the extracts. All the prepared extracts were stored at 4 °C until analysis.

### 2.2. Reagents and Chemicals

Standard AED diazepam (DZP) and Pentylenetetrazol (PTZ) were commercially obtained from Sigma Aldrich (USA). TRIzol^®^ reagent was purchased from Invitrogen, USA. The primer sets used for QuantiTect SYBR Green dye (Qiagen, Valencia, CA, USA) were as follows: NF-κB: Dr_nfkb1_2_SG QuantiTect Primer Assay (Cat no. QT02498762); BDNF: Dr_bdnf_1_SG QuantiTect Primer Assay (Cat no. QT02125326); eef1a1b: Dr_eef1a1b_2_SG QuantiTect Primer Assay (Cat no. QT02042684); c-Fos: Dr_fos_1_SG QuantiTect Primer Assay (Cat no. QT02103243).

### 2.3. Animals Studies

The zebrafish (Danio rerio) used in this study were of heterogeneous wild-type strain, with a short-fin phenotype (aged 3–4 months). The zebrafish were purchased from an aquarium in Subang Jaya, Malaysia. Both males and females were used in this study, as there are no reported sex-specific differences in response to PTZ and Kava extract. These zebrafish were maintained at the Monash University Malaysia Animal Facility under recommended standard husbandry conditions. The fish tanks were filled with water (pH 6.9–7.1) and maintained at a temperature around 28 °C. The zebrafish were provided illumination with a 250-lux light set into a 14:10 h light: dark cycle. The zebrafish received TetraMin^®^ Tropical Flakes three times a day, through an automated feeder. A total of 125 zebrafish were used in this study, 35 fish for the toxicity test (*n =* 7 for 5 different concentrations) and 90 for the anti-convulsant study (*n =* 10 for each study group). All animal experiments reported in this study were approved by the Monash University Malaysia Animal Ethics Committee (MUM-AEC).

### 2.4. Toxicity Screening

The toxicity study of the prepared extracts of Kava was carried out as per the Fish, Acute Toxicity Test provided by OECD Guidelines for the Testing of Chemicals (Section 2 from Test No. 203) [11]. Each treatment group would have seven zebrafish (*n =* 7) placed separately in tanks with 1L capacity each with 100 mg/L of the Kava extracts. The aqueous Kava extracts prepared were from stem peels (SPA), stems with no peels (SNPA), and whole stems (SWA), respectively. The zebrafish were examined every 15 min for the initial 2 h of exposure, and every 30 min thereafter, for the first day. Subsequently, they were checked three times a day up till 96 h. Additional criteria applied during the study were: (i) if any zebrafish showed signs and symptoms of severe pain or suffering, they were euthanized with benzocaine overdose; (ii) if there was at least one death, the concentration would be decreased by a factor of two and the protocol repeated; (iii) the “high” dose would be the concentration without any deaths of zebrafish, and the “low” dose would be half the concentration of the “high” dose. 

### 2.5. Behavioral Study

In total, there were nine groups (*n =* 10) of zebrafish. Zebrafish of three months’ age were selected with a weight range between 0.4 to 0.8 g. The Kava extracts and diazepam (DZP) were dissolved in the tank water while distilled water was used for preparation of pentylenetetrazole (PTZ) solution for intraperitoneal injection. The groups and treatments selected were as follows:

Group I: PTZ (170 mg/kg) used as negative control;
Group II: tank water used as vehicle control;
Group III: DZP (10 mg/L) + PTZ (170 mg/kg) used as positive control;
Group IV: Kava extract SNPA (100 mg/kg) + PTZ (170 mg/kg) used as treatment group 1;
Group V: Kava extract SNPA (50 mg/kg) + PTZ (170 mg/kg) used as treatment group 2;
Group VI: Kava extract SPA (50 mg/kg) + PTZ (170 mg/kg) used as treatment group 3;
Group VII: Kava extract SPA (25 mg/kg) + PTZ (170 mg/kg) used as treatment group 4;
Group VIII: Kava extract SWA (50 mg/kg) + PTZ (170 mg/kg) used as treatment group 5;
Group IX: Kava extract SWA (25 mg/kg) + PTZ (170 mg/kg) used as treatment group 4.

### 2.6. PTZ-Induced Seizure Model

For incubation of the zebrafish, 1 L treatment tanks were used, and the incubation was carried out for 45 min before PTZ administration. For temporary anesthesia, the zebrafish were captured by use of a fish-holding net and the captured zebrafish were transferred to a 30 mg/L Benzocaine solution. Once anesthetized, each zebrafish was weighed using an electronic balance, to calculate the injection volume based on the PTZ dose of 170 mg/kg. In order to restrain the zebrafish during the intraperitoneal injection, a soft sponge (soaked in water) with a 10 mm incision depth was used and placed on a 60 mm Petri dish. The injection was administered in a region below the pelvic girdle via the abdominal cavity. The injection volume was fixed at 10 µL per 1 g body weight. Post intraperitoneal injection, the fish were immediately transferred to a 13 L observation tank. All study groups of zebrafish were administered 170 mg/kg PTZ, except for vehicle control, which was substituted with tank water.

The swimming pattern of the PTZ-induced zebrafish was recorded for a duration of 10 min with a video recorder, after anesthesia recovery. The determination of the highest seizure score and the onset was based on the recorded videos. The Smart tracking software was used to analyze the swimming patterns. The scoring system used, is as shown below [12]:

Score 1 - Short swim indicates swimming mainly at the bottom of the tank;
Score 2 - Increased swimming activity and high frequency indicates opercular movement;
Score 3 - Indicates burst swimming, left and right movements, as well as erratic movements;
Score 4 - Indicates circular movements.

### 2.7. Gene Expression Study

After recording the zebrafish behavior for 10 min, the brain tissues were harvested and placed into 200 µL of TRIzol^®^ and stored immediately at −80 °C. The mRNA was extracted using chloroform and isopropanol as described by Brandon et al. [13]. The isolated mRNA was then quantified with NanoDrop Spectrophotometer and converted to cDNA, following the protocol provided by the manufacturer (QuantiTect^®^ Reverse Transcription Kit).

The mRNA expression was determined using the StepOne^®^ real-time PCR. Three genes investigated in this study were c-Fos, Nuclear Factor Kappa-light-chain-enhancer of activated B cells (NF-κB), and Brain-Derived Neurotrophic Factor (BDNF), and Elongation factor 1-alpha-1b (eef1a1b) was selected as the housekeeping gene. The rt-PCR protocol provided in the QuantiText SYBR Green dye manual was used. The relative expression was calculated using the formula: 2 ^ (housekeeping − gene of interest), post calculation of the threshold cycle (Ct) values.

### 2.8. Data Analysis

GraphPad Prism software was used to perform statistical analysis. The data were analyzed with a One-way Analysis of Variance (ANOVA), and mean ± standard error of the mean (SEM) were calculated. In addition, Tukey’s test was used for multiple comparisons and Post-Hoc analysis. All the other treatment groups were compared with the negative control. Zebrafish swimming patterns were tracked using the Smart tracking software (Pan Lab, Harvard apparatus V3.0.05). The Applied Biosystems StepOnePlus^TM^ Real-Time PCR System was used for gene expression analysis.

## 3. Results

### 3.1. Toxicity Study

There have been no published studies that report the evaluation of safety/toxicity of Kava stems extracts, particularly in zebrafish. Thus, in this study, before evaluating the biological activity of these extracts, a toxicity study was carried out. As this is a novel study on the anti-convulsive potential of the Kava stems extracts, the determination of safe doses was essential. The OECD guidelines specified that a 100 mg/L limit test on seven zebrafish would yield a 99% confidence level and that the LC_50_ would exceed 100 mg/L if no deaths occur in the span of 96 h [1].

In the limit test of 100 mg/L Kava extract, two extracts SPA and SWA had one mortality each, after overnight exposure. There was no mortality observed with 100 mg/L dose of SNPA. The whole procedure was repeated with another group (*n =* 7) for half of the concentration, 50 mg/L of SPA, and SWA. There were no mortalities thereafter. Therefore, the “high” dose for SNPA was set at 100 mg/L and the “low” dose as 50 mg/L. For SPA and SWA, the “high” dose and “low” dose were set as 50 mg/L and 25 mg/L, respectively.

### 3.2. Behavioral Study

#### 3.2.1. Swimming Pattern

**Figure 1:**
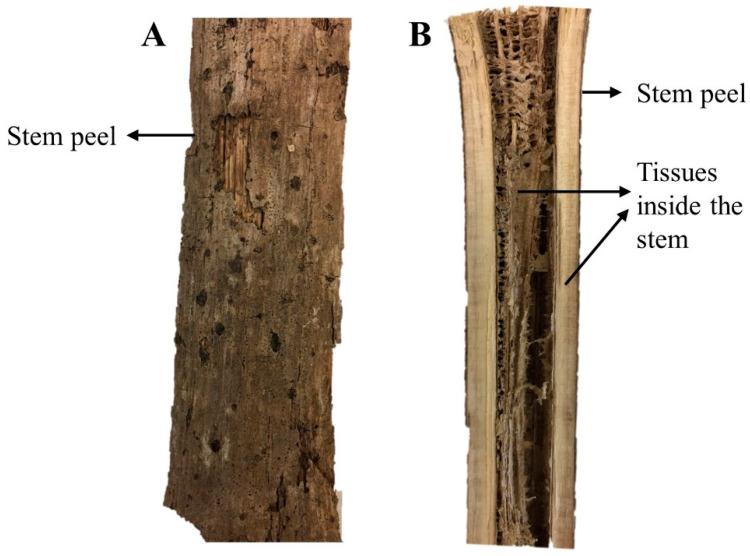
Pictorial representation of the stems of *Piper methysticum,* (**A**) longitudinal view of the outer part of stem, (**B**) longitudinal view of the inner tissues of stems.

**Figure 2:**
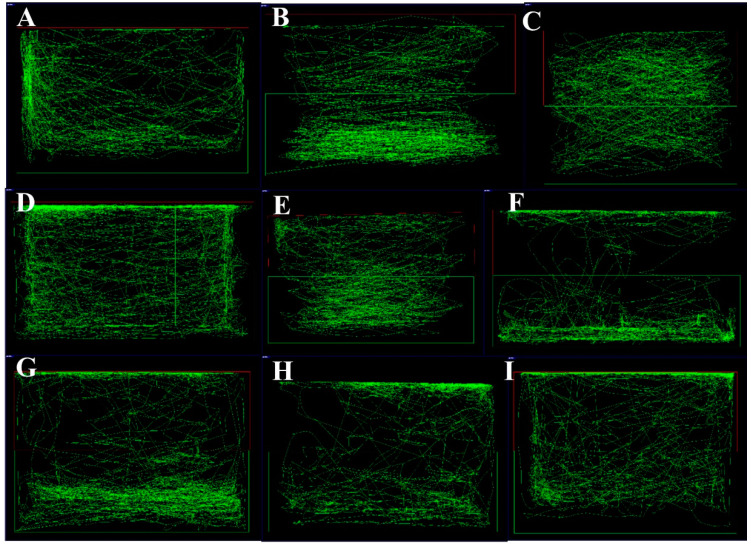
Representative images of zebrafish swimming pattern for various groups tested for behavioral effects of *Piper methysticum* stem extracts: (**A**) pentylenetetrazol (PTZ) (170 mg/kg) − negative control, (**B**) diazepam (DZP) (10 mg/L) + PTZ (170 mg/kg) − positive control, (**C**) vehicle control, (**D**) stems with no peels (SNPA) (100 mg/kg), (**E**) SNPA (50 mg/kg), (**F**) whole stems (SWA) (50 mg/kg), (**G**) SWA (25 mg/kg), (**H**) stem peels (SPA) (50 mg/kg), (**I**) SPA (25 mg/kg).

In Figure 1, a pictorial representation of Kava stem tissues is provided. The extracts of these stems as detailed above were tested for their effects on swimming patterns of zebrafish. Representative images of the swimming pattern of the zebrafish recorded 10 min after the administration of PTZ (170 mg/L) are shown in Figure 2A–I. The negative control received PTZ challenge and showed an erratic swimming pattern in circular movements, especially at the sides and the bottom of the tank. In the positive control, the zebrafish were pre-treated with diazepam before the PTZ challenge. The pre-treated zebrafish exhibited more normal swimming behavior, which is characterized by a more spread out pattern throughout the whole tank. They spent an equal amount of time and distance on the top and bottom half of the tank. The vehicle control group showed a normal swimming pattern with a slight preference of swimming at the bottom half of the tank. When treated with the extracts, all except 50 mg/L of SNPA showed an erratic swimming pattern, where most of the time was spent at the top and bottom of the tank only. After treating with 50 mg/L of SNPA, it was observed that the swimming pattern fitted a profile leaning towards the positive and vehicle control groups, where only zig-zag movements were observed.

#### 3.2.2. Determination of Seizure Score and Onset Time

**Figure 3:**
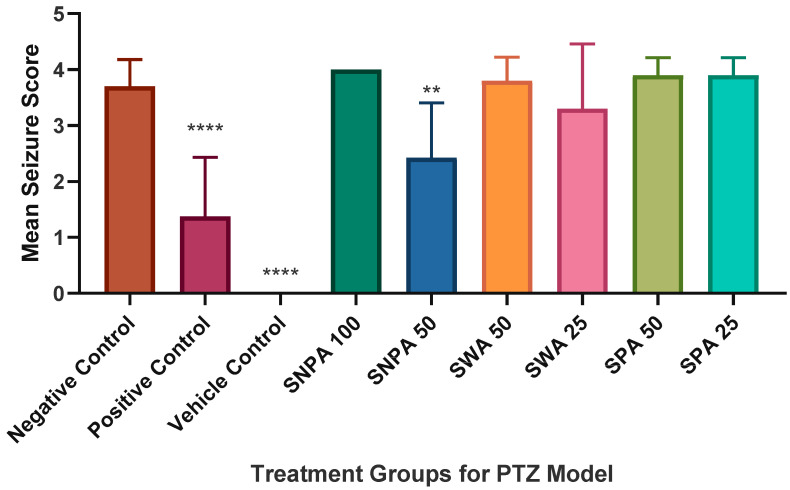
Graphical representation of mean seizure score in various groups of PTZ model. The data was compared to the negative control group using one-way ANOVA (**** *p* < 0.0001; ** *p* < 0.01). The figure represents mean ± SEM of the highest seizure score in each group (*n =* 10).

**Figure 4:**
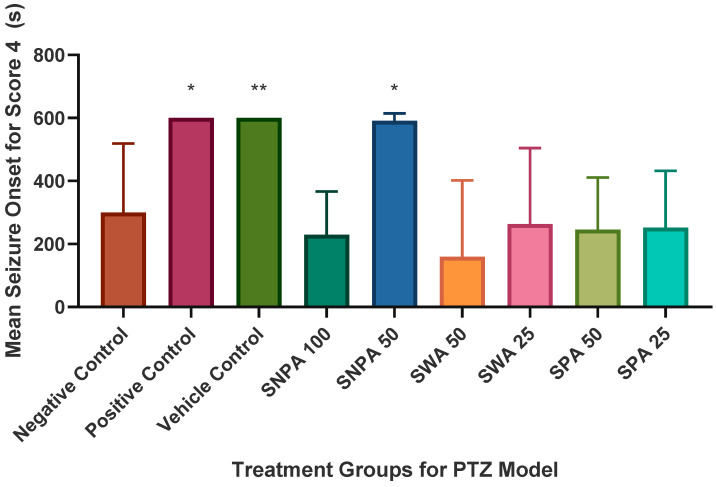
Graphical representation of mean seizure onset for score 4 in various groups of PTZ model. The data was compared to the negative control group using one-way ANOVA (* *p* < 0.05; ** *p* < 0.01). The figure represents mean ± SEM of seizure onset time (*n =* 10).

When challenged with PTZ, the overall seizure score was 3.70, as displayed in Figure 3. There was a significant reduction after pre-treatment with diazepam which produced a mean score of 1.38 (**** *p* < 0.0001). No seizures were observed in the vehicle control group. With 45 min pre-treatment with 50 mg/L of SNPA, the mean seizure score was significantly reduced to 2.43 in this group compared to the negative control (** *p* < 0.01). Other treatment groups were not found to have any statistical significance in terms of the mean seizure score. This trend was similarly reflected in the onset time for seizure score 4, as shown in Figure 4. The onset time was significantly increased from 300.3 s in the negative control group, which received only PTZ challenge, compared to 591.4 s in the treatment group which was pre-treated with 50 mg/L of SNPA (* *p* < 0.05). 

**Figure 5:**
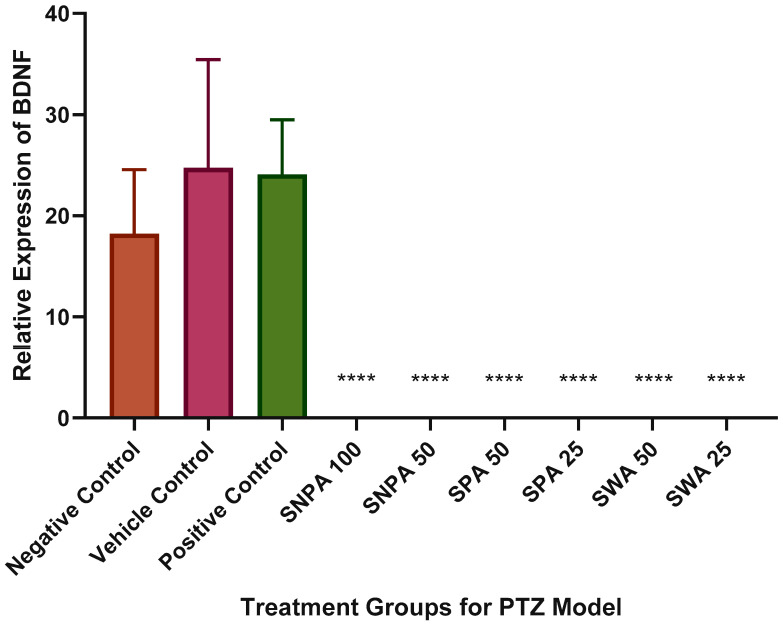
Graphical representation of the relative expression of brain-derived neurotrophic factor (BDNF) in treatment groups (*n =* 6). The data were expressed as mean ± SEM by applying one-way ANOVA (**** *p* < 0.0001).

There were no significant differences found in the relative expression of BDNF among negative control, vehicle control, and positive control group as shown in Figure 5. Nevertheless, the vehicle control and diazepam treated group showed higher expression levels of BDNF, compared to the negative control group. The relative expression of BDNF was significantly decreased in all Kava extract treated groups.

**Figure 6:**
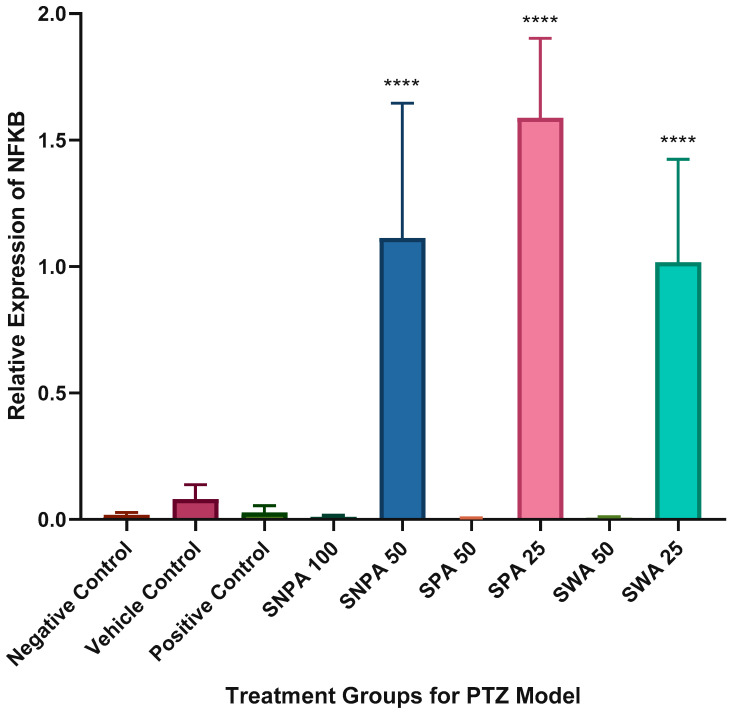
Graphical representation of the relative expression of NF-κB in each treatment group (*n =* 6). The data were expressed as mean ± SEM using one-way ANOVA (**** *p* < 0.0001).

A significant increase was observed in the relative expression of NF-κB in groups treated with “low” doses of Kava stems extract, compared to the negative control. The groups treated with “high” doses showed a decreasing trend in relative expression of NF-κB. There was no significant difference observed between the positive and negative control groups, but there was a slight increase in the NF-κB expression in the group treated with diazepam (as indicated in Figure 6).

**Figure 7:**
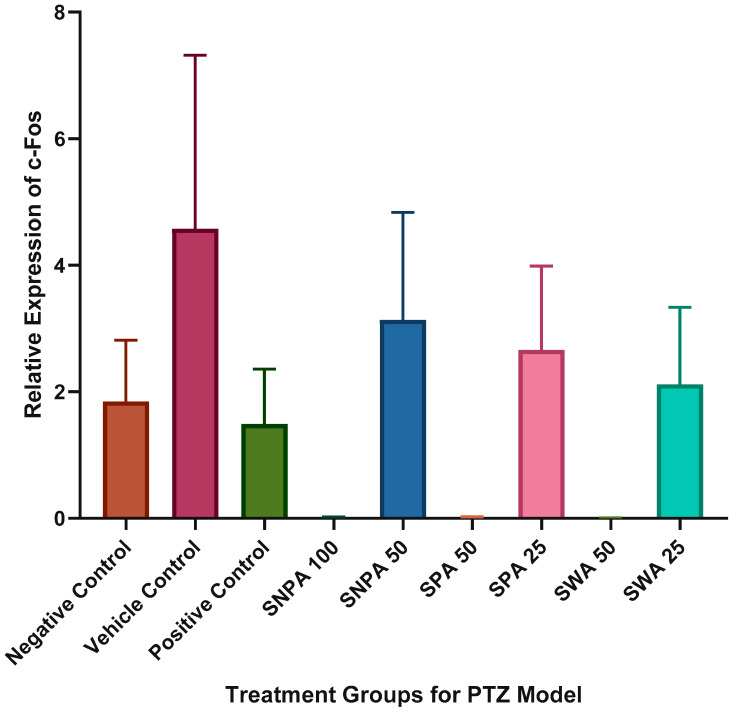
Graphical representation of the relative expression of c-Fos in treatment groups (*n =* 6). The data were expressed as mean ± SEM using one-way ANOVA.

No statistical significance was observed in the relative expression of c-Fos in all treatment groups (Figure 7). The diazepam treated group exhibited a slight reduction in c-Fos expression compared to the PTZ treated group. The expression levels of c-Fos were found to be much higher in the vehicle control group, and the “low” dose treated group, but not significant at a level of α = 0.05.

## 4. Discussion

In this study, we investigate the toxicity and anti-convulsive properties of extracts of various stem tissues of Kava. The study provides new findings through a detailed investigation of extracts prepared from carefully selected plant tissues, which could have the potential for enhancing efficacy or cause toxicity of a popular herb and have not been studied to date. In reports published by Jhoo et al., in vitro cytotoxicity of non-polar fractions of Kava extracts prepared from roots, leaves, and stem peelings were observed in human hepatoma HepG2 cells. The studies report Flavokavain B as the compound responsible for hepatotoxicity [14,15]. Pipermethystine, which is a major alkaloid found in the Kava leaves and stem peelings, is reported to cause in cell death in HepG2 cells via mitochondrial disruption. It is suggested to be the contributory compound in hepatotoxicity [16,17]. These findings corroborate with the SNPA extract causing no deaths in zebrafish in the toxicity studies, as these extracts were devoid of stem peelings. 

In the group that received 50 mg/L of SNPA pre-treatment, the behavior of bottom-dwelling was reversed, similar to the group that was treated with diazepam. The seizure score and onset time was also significantly reduced. Bottom-dwelling is a sign of anxiety that can be observed in zebrafish that was newly transferred to a new tank [18]. It also resembled stupor-like behavior associated with the epileptic condition after inducing seizures with PTZ [12]. Parker et al. report that bottom-dwelling is reduced after the administration of anxiolytics. The reduction in the bottom dwelling after treatment with SNPA extract indicates the anxiolytic properties of this extract of Kava stems [19]. Kavain (one of the major kavalactones) was found to have direct interaction with a subtype of GABA_A_ receptors at a site that was distinct from the classical binding site of benzodiazepines [8]. This may be the contributing factor to the anti-convulsive properties of SNPA as PTZ is an agonist of the GABA_A_ receptor, which could induce seizures by modulating the GABAergic inhibitory action [20]. 

The brain-derived neurotrophic factor (BDNF) is reported to play a vital role in epileptogenesis condition [21]. The upregulation of BDNF mRNA was reported in chemical-induced seizures [22]. All treated groups, except the negative control, exhibited a significant decrease in the relative expression of BDNF. However, the pre-treatment with diazepam exhibited no reduction in the BDNF expression. This effect was opposite to the effect reported by Kornblum et al., where the diazepam treatment impeded the elevation of BDNA mRNA [23].

The expression levels of NF-κB were significantly increased after inducing seizures with PTZ in rats (Prasad, Pilcher and Joseph, 1994). However, this was not observed in the negative control group as shown in Figure 5. A plausible reason could be that the NF-κB expression increased gradually in the span of 24 h before reaching the maximum expression level [24]. The higher expression levels of the 50 mg/L SNPA pre-treated group suggest that it may reduce the convulsive potential by modulating other pathways independent of NF-κB.

The relative expression of c-Fos also showed no statistical significance, which is in agreement with the findings reported by Choo et al. [24]. One of the possible explanations for this effect is that c-Fos require approximately 30 min to build up a significant difference in the expression levels after administration of a chemical convulsant [25]. 

Studies report that kava root extracts exhibit anticonvulsant effect by enhancing binding of (GABA) type A receptors and ligands [26,27]. These effects are suggested to be due to the presence of Kavalactones such as Kavain, desmethoxyyangonin (DMY), and 7,8-dihydromethysticin (DHM). These kavalactones are also abundantly present in the stems and aerial parts of the Kava plant including the leaves. The difference between constituents of the stems and roots lies in the presence of higher content of Pipermethystine in the stems and leaves, compared to the roots. However, to the best of our knowledge, not anticonvulsant effects but hepatotoxic effects have been suggested for Pipermethystine. Thus, we suggest that the anticonvulsant effect of extracts of various tissues of Kava stems may be due to presence of kavalactones.

The aqueous Kava extract from the stems without peel (SNPA) at the dose of 50 mg/L exhibited anti-convulsive potential after pre-treatment of 45 min. This effect is comparable to the therapeutic effects of diazepam in terms of seizure intensity and onset time. The expression of BDNF was significantly reduced after treatment with the aqueous Kava extracts, which may be the contributing factor to its anti-convulsive properties.

## 5. Conclusions

The study demonstrates that the peeled stem of *Piper methysticum* can serve as a novel and promising candidate for the design of anti-convulsive drugs. The findings encourage future studies to investigate the modulatory effects of peeled Kava stem extracts on the genetic mechanisms altered by the prepared extracts.
